# Assessment of the Effects of Salt and *Salicornia herbacea* L. on Physiochemical, Nutritional, and Quality Parameters for Extending the Shelf-Life of Semi-Dried Mullets (*Chelon haematocheilus*)

**DOI:** 10.3390/foods11040597

**Published:** 2022-02-18

**Authors:** Hee-Geun Jo, Ramakrishna Chilakala, Min-Ju Kim, Yong-Sik Sin, Kyoung-Seon Lee, Sun-Hee Cheong

**Affiliations:** 1 Department of Marine Bio-Food Sciences, Chonnam National University, Yeosu 59626, Korea; altkwh@naver.com (H.-G.J.); ramach2006@gmail.com (R.C.); modori96k@naver.com (M.-J.K.); 2 Department of Environmental Engineering & Biotechnology, Mokpo National Maritime University, Mokpo 58628, Korea; ekdmarhf@naver.com (Y.-S.S.); kslee@mmu.ac.kr (K.-S.L.)

**Keywords:** salted semi-dried mullet, *Salicornia herbacea* L., quality, nutritional characteristics

## Abstract

Mullet, a coastal fish species, is commonly used as a salted dried fish in many countries, including Korea, Japan, and the southeastern United States. The purpose of this investigation was to develop high-quality products of salted semi-dried mullet (SSDM) using natural salt and *Salicornia herbacea* L. (SAL). The antioxidant activity of SAL was investigated by in vitro studies. The physicochemical and nutritional characteristics of fresh mullet (FM), salted control (SSDM-CON), and SAL-treated (SSDM-SAL) mullet groups were analyzed. The moisture, ash, and crude protein contents were significantly increased in the SSDM-SAL group, whereas the salinity was decreased when compared with the SSDM-CON group. Lipid oxidation occurred in the FM and SSDM groups, as indicated by the increase in peroxide (PV), acid (AV), and thiobarbituric acid reactive substance (TBARS) values during the storage period. The protein pattern on the sodium dodecyl-sulfate polyacrylamide gel electrophoresis (SDS-PAGE) analysis showed similarities between the groups, while the amino acid and fatty acid contents also varied in the FM and SSDM groups depending on their processing methods. Initially, the total bacterial count was significantly higher in the SSDM groups than in the FM group. However, the SSDM-SAL group had a markedly lower total bacteria count than the FM and SSDM-CON groups during 21 days of refrigerated storage. This result indicates that SAL treatment can improve mullet’s safety from microorganisms, includes beneficial biochemical parameters, and can extend their shelf-life through refrigerated storage.

## 1. Introduction

Mullet is a species of fish that is found all over the world. It can be categorized into gray mullet (*Mugil japonicus*) and red mullet (*Mullus*) [1,2]. Mullets are commercially important, since they contribute to large estuarine and coastal fisheries in many countries, especially in the Mediterranean. The overall production of mullets represents 2.6% of total marine aquaculture production [3]. Gray mullet production increased in the last decade, from 101,182 tons in 2008 to 130,233 tons worldwide in 2018. Egypt is the main producer, followed by the Republic of Korea, Italy, Taiwan, and Israel [4,5]. Fish provides optimal nutrients that are essential for human growth and development; fish meat is known to be high in protein content and also contains eicosapentaenoic (EPA, 20: 5n-3,) and docosahexaenoic (DHA, 22: 6n-3,) fatty acids, which are essential for humans [6]. Fatty acids are involved in various physiological processes that regulate homeostasis, host defense, organ function, and immune and inflammatory responses [7]. Increasing levels of aquaculture production has led to growing interest in the development of preservation techniques without nutrient loss. However, fish products contain a high moisture percentage, which makes them spoil more easily; moreover, the rapid impairment of fish quality after harvest is mainly due to microbial spoilage and fatty acid oxidation, which reduces the meat’s shelf-life [8]. This easy rancidity makes fish products difficult to process, store, and distribute.

Food materials containing fats and oils are acidified via auto-oxidation in the presence of oxygen and heat, thus affecting food quality [9]. Acidification is a major contributor to product deterioration, causing economic losses of more than $700 million annually [10]. These lipid oxidized products not only lose their nutritional values but also show reduced consumer palatability, making them unsuitable for human consumption. In addition, various radicals generated by lipid oxidation and secondary products such as carbonyl compounds can cause aging, carcinogenesis, and atherosclerosis [11].

Freezing and drying are the most widely used fish processing methods for preservation due to their advantage of providing microbiological safety. However, in the freezing process, products easily crumble. Excessive drying has a disadvantage in that the texture is degraded and the quality deteriorates due to the oxidation of fatty acids and browning [12]. Several studies have pointed out that proximate and lipid compositions, specifically fatty acid contents, can affect food processes and change the nutritional values of processed raw samples. However, different processing methods show large variations in individual fatty acid contents [13]. To solve this problem in Korea, the intestines and gills of fish are removed by abdominal incision before the fish are treated with salt followed by sun-drying for one day [14]. Recently, to compensate for the shortcomings of dry products, interest has grown in salted semi-dried products, which have a slightly higher moisture content than dry products, and thus a more desirable flavor, texture, and shelf-life. Despite the essential functions that sodium provides when producing high-quality and safe meat products, the high level of sodium intake from processed foods can increase the risk of hypertension and cardiovascular diseases [15].

*Salicornia herbacea* L. (SAL) is known to grow in wetlands or mud on the coast of Korea. It is rich in potassium and calcium, possesses antioxidant activity and has triglyceride and cholesterol reducing effects. It may help to prevent or preserve the lipid oxidation of aquatic foods. In addition, it is effective as a folk remedy for various diseases, such as asthma, high blood pressure, cancer and diabetes [15]. SAL is also used as a seasoning and/or a salt replacer when processing foods such as steamed rice cake, tofu, kimchi, cooked sausages, beef jerky, and so on [15,16,17]. Therefore, the aim of this study was to investigate chemical changes that occur during the refrigerated storage of SAL-treated semi-dried mullets. The concentration of SAL was chosen based on the antioxidant activity on DPPH (2,2-diphenyl-1-picrylhydrazyl) and ABTS (2,2′-azino-bis (3-ethylbenzthiazoline-6-sulfonic acid)) radical analysis by in vitro studies, and the preferred SAL concentration was used for mullet processing and preservation over 21 days. In addition, we investigated SAL’s protective effect against microorganisms and its extension of shelf-life by protecting physicochemical, nutritional, and quality parameters. Accordingly, salt content, moisture, water activity, crude fat and protein contents, and meat color were analyzed. Moreover, we studied its effect on the fatty acid oxidation and rancidity of mullet by monitoring the peroxide, acid values, and thiobarbituric acid levels in the SAL-treated group. Fatty acid, amino acid and protein deterioration, along with the microorganism content, were determined in the fresh (FM), salted semi-dried (SSDM-CON), and SAL-treated (SSDM-SAL) mullet groups during refrigerated storage.

## 2. Materials and Methods

### 2.1. Preparation of the Sample

The natural liquid extract from *S. herbacea* L. (without water content) was obtained from the Shinanharmcho company (Shinanharmcho.com) in Gwangju, South Korea. Gray mullets with an average weight of 1.14 ± 0.09 kg and a length of 56.05 ± 2.62 cm were used in this study. They were supplied in September, 2020 by the Fishery Union Shinan Geonjeong company in Sinan, South Korea. The blood and other mullet wastes were removed via abdominal incision. The mullets were then washed with tap water for 30 min and soaked for 2 h in a 3% sea salt solution for the SSDM-CON group or soaked in a glasswort solution (2.8% sea salt + 0.2% SAL extraction) for the SSDM-SAL group. Subsequently, the samples were air-dried in a chamber for 36 h at 20 °C, packed individually in a sterile sampling bag (Labplas Twirl EM^TM^), then refrigerated in storage at 4 °C for 21 days. Each treatment was applied to one fish, which was used for five replicate analyses in each experimental group.

### 2.2. Anti-Oxidant Activity of Salicornia herbacea L.

To evaluate antioxidant activity by DPPH and ABTS radicals’ scavenging ability, liquid extractions of SAL samples were diluted in concentrations of 0.015, 0.03, 0.06, 0.125, 0.5, 1, 2, and 4; then, 0.2 mM DPPH (2,2-diphenyl-1-picrylhydrazyl) was added 1:1, followed by the reaction at room temperature for 30 min. The absorbance was measured at 517 nm [18]. For the ABTS radical, 7.4 mM ABTS was prepared using a PBS buffer, 2.6 mM The potassium persulfate was prepared and mixed 1:1, and then kept in a dark place at room temperature for one day. When the mixed ABTS and PBS were combined in a ratio of 1:15 and measured by absorbance at 734 nm, they became 0.7. The SAL sample was diluted by concentration, the reagent and the sample were mixed in a ratio of 950:50, and then reacted in a dark room for 30 min at room temperature. The absorbance was then measured at 734 nm [18]. The radicals’ scavenging ability (%) was calculated using the following Equation (1).

% inhibition = 100 (Abs_control_ − Abs_sample_)/Abs_control_ (1)

### 2.3. Proximate Composition, Salinity and Water Activity (a_w_)

Proximate compositions, including the salinity and water activity of the mullets, were analyzed using authorized protocols from the Association of Official Analytical Chemists [19]. Minced whole-fish samples were used to analyze the proximate compositions. The moisture contents were measured by drying the samples at 105 °C to reach constant weights. Protein levels were analyzed using the Kjeldahl procedure suggested by AOAC methods [20]. In the process, approximately 1 g of raw material was hydrolyzed with 15 mL concentrated sulfuric acid (H_2_SO_4_) containing two copper catalyst tablets in a heat block (Kjeltec system 2020 digester, Tecator Inc., Herndon, VA, USA) at 420 °C for 2 h. After cooling, H_2_O was added to the hydrolysates before neutralization and titration. The amount of total nitrogen in the raw materials was multiplied with both the traditional conversion factor of 6.25 and species–specific conversion factors to determine the total protein content [21].

The total lipids were analyzed using the Soxhlet method described by AOAC [22]. In total, 10 g of samples were analyzed using 300 mL of a diethyl ether and petroleum ether mixture (1:1) in Soxhlet apparatus (VWR international, Fontenay-sous-Bois, Rue Carnot, France) for 6 h. The solvent was removed using a BÜCHI Rotavapor R-144 rotary evaporator (BÜCHI Sarl, Villebon-sur-Yvette, Av. du Québec, France) at 40 °C. The vacuum was interrupted by introducing N_2_ (g) (inert atmosphere) to avoid any oxidative damage to the lipids caused by exposure to air. The residue remaining as deposits on the flask wall was collected with a spatula. The residue yields were calculated and expressed based on the initial mass of the sample. A muffle furnace was used to measure the ash content after the sample incineration at 550 °C. For the salinity measurement, deionized water (five times the volume of the sample) was added to each sample. After stirring and centrifugation, each sample was filtered to obtain a filtrate and analyzed using a salinity meter (PAL-ES, ATAGO, Tokyo, Japan). Water activity was measured at 27 °C using an electric hygrometer (Hygrodynamics, Inc., Silver Spring, MD, USA).

### 2.4. Chromaticity

The chromaticity of the lateral-side white muscular parts of the FM and SSDM samples were measured with a color meter (ZE2000, Nippon Denshoku Co., Tokyo, Japan) using the CIE L*a*b* system [23]. During this process, lightness (L*) values for black (L* = 0) and white (L* = 100) were determined; positive a* values represented the redness, and a negative a* value represented the greenness; positive b* values represented the yellow, and a negative b* value represented the blue. A standard black and white ceramic tile was used to calibrate the instrument before every measurement. The color measurements were performed at room temperature. The mean values were obtained from five measurements for each experiment.

### 2.5. Lipid Oxidation

The lipid oxidation of the FM and SSDM groups were evaluated according to peroxide (PV), acid (AV), and thiobarbituric acid reactive substance (TBARS) levels. PV was analyzed following the procedure described by Egan et al. [24]. In this process, 0.5 g of freeze-dried SSDM sample ground powder was dissolved in 25 mL of chloroform (3:2, *v*/*v*) containing acetic acid, then 1 mL of saturated potassium iodide was added before being kept in a dark place for 10 min. Next, 30 mL distilled water and 1 mL of starch solution (1% *w*/*v*) were added, and the total mixture solution was titrated against sodium thiosulfate (0.01 N) until the blue color disappeared. PV values are expressed as milliequivalents of peroxide oxygen per kg of sample (mEq/kg). AV was calculated using the titration method described by Pearson [25]. In this process, 1 g of extracted oil was dissolved into an equal volume of indicator, which contained diethyl ether, ethanol and a 1% phenolphthalein reagent. This final solution was titrated against 0.1 M NaOH. TBARS levels were assessed to measure lipid peroxidation following the modified method as described by Faustman et al. [26]. In this process, 20 g each of the FM and SSDM samples were homogenized for 15 s with 50 mL distilled water containing trichloroacetic acid (final concentration: 15%) and centrifuged at 33,540× *g* for 5 min. The supernatant part was then collected after filtering with Whatman No. 1 filter paper. Next, 8 mL of the supernatant part was added with 2 mL of thiobarbituric acid (0.06 M); the mixture was vortexed for 15 s, heated at 95 °C for 1 h, and cooled on an ice tray. The sample absorbance value was measured at 523 nm using a UV–vis spectrophotometer and the results were expressed as mg MDA/kg of the sample.

### 2.6. Fatty Acid Analysis

The total lipid content was extracted from the FM and SSDM samples by adding a chloroform and methanol mixture containing 0.01% of butylated hydroxytoluene at a 2:1 ratio (*v:v*). The obtained lipid contents were dried using a vacuum rotary evaporator (VV 2011, Heidolph Co., Ltd., Schwabach, Germany). These lipids are transformed into fatty acid methyl esters (FAMEs) via base-catalyzed trans-esterification in the presence of sodium methoxide at 30 °C for 2 h [27]. These FAMEs were measured using gas chromatography (Shimadzu GC-17A, Shimadzu, Tokyo, Japan), which is fused with a silica capillary column (SP^TM^-2560, 100 m × 0.25 mm i.d, 0.25-μm film thickness, Supelco, Bellefonte, PA, USA). To perform the fatty acid analysis, 1 μL of the FAMEs n-hexane sample was injected into the column. The initial isothermic period was maintained for 10 min at 140 °C. This temperature was increased by 4 °C/min up to 240 °C and the isothermic period was maintained for 30 min. The injection and detector ports were maintained at a 260 °C temperature using helium gas. The FAME peaks’ retention time was compared with standards used for identifying fatty acid compositions (47885-U, Supelco 37 Component FAME Mix, Supelco, Sigma-Aldrich, Bellefonte, PA, USA). The results of the fatty acid compositions in each experimental group were expressed as g/100 g total fatty acids.

### 2.7. Amino Acid Analysis

For measuring the amino acids, 80 mg of sample was dissolved with 10 mL of 6 N HCl solution in a test tube and purged with nitrogen (N_2_) gas. The product was then hydrolyzed at 110 °C for 24 h in a dry oven and added to a sodium-distilled buffer (pH 2.2) [28]. The mixture was filtered using a syringe filter (0.45 μm). The amino acids were analyzed by ninhydrin reaction using a Biochrom 20 amino acid analyzer (Pharmacia Biotech, Cambridge, UK). The amino acid compositions were measured at 440 and 570 nm absorbance.

### 2.8. SDS-PAGE Analysis

The protein profile was analyzed for each SSDM sample using SDS-PAGE. Briefly, 0.1 g of the sample was homogenized with 500 μL of lysis buffer. After centrifugation at 12,000× *g* for 30 min, the supernatant part was collected. For the protein quantification, the sample buffer was added into the supernatant and heated at 100 °C for 5 min. Next, 10–15 μL sample was subjected to SDS-PAGE analysis using a Mini PROTEAN Tetra cell (Bio-Rad Lab., Inc., Hercules, CA, USA) for 90 min according to Laemmli [29].

### 2.9. Microbiological Analysis

Commercially available 3M™ Petrifilm™ plates (3M Microbiology Products, St. Paul, MN, USA) were used to conduct the total microbiological analysis, following the manufacturer’s instructions. In this process, 10 g of FM and SSDM samples were homogenized with 100 mL of 0.85% physiological saline for 2 min in a sterilized sample bag (3M^TM^ Sample Bag). Next, 1 mL of the homogenized sample suspension was cultured using 3M™ Petrifilm™ plates at 35 ± 1 °C for a 24 to 48 h incubation period [30]. Most bacterial species produced different colonies, with different colors. The red-colored colonies developed on the 3M™ Petrifilm™, which are visible to the naked eye, and the number of colonies on each 3M™ Petrifilm™ were counted. The average number of red-colored colonies was expressed as log cfu/g after being multiplied by the dilution factor used for counting total microorganisms. The same protocol was used to measure the Coliforms, *Escherichia coli* and *Staphylococcus* followed by the 3M™ Petrifilm™ plate counting method, according to the manufacturer’s instructions. The visible, red-colored colonies surrounded by trapped gas indicated Coliform bacteria, while *E. coli* appeared as blue-colored colonies with trapped gas on the 3M™ Petrifilm™. However, red-violet colonies appeared on the *Staphylococcus* analysis, and, depending on the test sample, black and green colonies also appeared, in addition to reddish-purple colonies. If black colonies appeared, definite *Staphylococcus* was considered, so the presence of a pink zone was confirmed by inserting a staph express disk (STX) for 1 to 3 h.

For the *Vibrio parahaemolyticus* analysis, 1 mL of diluted sample was inoculated into three petri dishes (0.3 mL, 0.4 mL, and 0.3 mL respectively) containing thiosulfate citrate bile salts sucrose (TCBS) agar medium, followed by incubation at 35 ± 1 °C for 24 h. Next, the number of visible cyan non-degradable colonies was counted for *Vibrio parahaemolyticus* analysis. The colony-forming unit (CFU) per gram of the sample was calculated and expressed as log cfu/g for the minimum limit for detection.

### 2.10. Statistical Analysis

All experiments were performed at least in duplicate and expressed by means ± standard deviations (SD). The statistically significant differences were considered at *p* < 0.05, using one-way analysis of variance (ANOVA), Duncan’s multiple range test and two-way for quantitative variable changes, which were being processed using the IBM SPSS statistic version 20 (Chicago, IL, USA).

## 3. Results and Discussion

### 3.1. Anti-Oxidant Activity of Salicornia herbacea L. (SAL)

**Figure 1:**
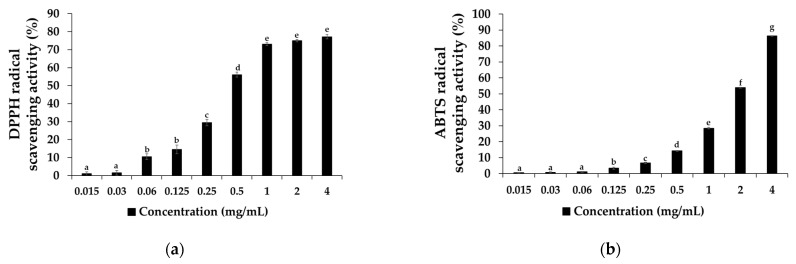
Antioxidant activity of SAL extraction on (**a**) 2,2-diphenyl-1-picrylhydrazyl (DPPH) free radicals and (**b**) 2,2′-azino-bis (3-ethylbenzthiazoline-6-sulfonic acid) (ABTS) radical scavenging activity (%). Values represent mean ± standard deviation (SD) (*n* = 5). Letters a, b, c, d, e, f and g in each bar graph represent significant difference at *p* < 0.05.

The effects of the SAL extract’s antioxidant activity on DPPH and ABTS radical results are presented in Figure 1; the in vitro results of the DPPH radical scavenging activity significantly increased in a concentration-dependent manner from the 0.06 mg/mL to 4 mg/mL extract concentrations, as shown in Figure 1a. Similarly, the ABTS radical scavenging activity experiment also significantly increased in a concentration-dependent manner, as shown in Figure 1b. It was established that SAL treatment increases the activity of scavenging DPPH and ABTS radicals, which reduced the lipid peroxidation and prevented the breakdown of biomolecules, as well as protected against microorganisms [31]. Moreover, the half-maximum inhibitory concentration (IC_50_) of the DPPH radical value was 0.44, while the ABTS radical IC_50_ value was 1.83, indicating that the SAL treatment inhibited the 0.44 to 1.83 mg/mL concentration more strongly for both DPPH and ABTS radicals. However, the antioxidant activity of SAL in the DPPH and ABTS radical assay indicates that the 2 mg/mL concentration had stronger free radical inhibition, of up to 60–75%, through donating electrons. Thus, a 2 mg/mL SAL concentration is able to reduce lipid peroxidation and extend mullet shelf-life during refrigerator storage due to scavenging ability. In addition, concentrations of more than 2 mg/mL of SAL also enhance the shelf-life by preventing the mullets from becoming rancid. However, concentrations of more than 2 mg/mL may influence the meat color, which is not suitable for consumer acceptance. In accordance with these results, we used the 0.2% SAL extraction for further treatment, along with salt, for mullet processing.

### 3.2. Proximate Composition, Salinity, and Water Activity (a_w_)

The results of the proximate composition, salinity, and water activity in the FM and SSDM groups are shown in Table 1. In the SSDM groups, the moisture contents were decreased more than in the FM group (70.6%). The moisture content in the SSDM-CON group (67.3%) was significantly (*p* < 0.05) lower than in the SSDM-SAL group (68%). Decreasing moisture content occurred during the dehydration of the sample. This can disrupt the muscle’s structure by denaturing sarcoplasmic and myofibrillar proteins. Similar observations have been reported when drying catfish and shrimps [32]. The SSDM groups had higher protein contents (24.59 to 25.68%) and ash contents (2.28 to 2.32) than the FM group. The ash contents of the FM and SSDM groups were significantly (*p* < 0.05) different as shown in Table 1. It has been reported that ash content is increased by the reduction in moisture during fish processing.

The SSDM-SAL group showed a significantly higher crude protein content (25.68%) than the FM and SSDM-CON groups, indicating that the nitrogen of the proteins during the drying period might have remained. The dry based (d.b) protein content results also indicated that the SSDM-SAL (80.32% d.b) group had higher amounts of protein than the FM (78% d.b) and SSDM-CON (75.22% d.b) groups. This demonstrates that the reduction in protein content in the SSDM-CON group might have been due to some nitrogen compound denaturation without 0.2% glasswort (SAL) treatment, thus decreasing the protein content and, consequently, the nutritional value of the meat. The SSDM-CON group had a higher crude fat content (3.24%) than the SSDM-SAL group (3.18%) and the FM group (2.83%). The dry base analysis results indicate that, higher amounts of crude fat were present in both the SSDM-CON (9.91%) and SSDM-SAL (9.94% d.b.) groups, compared to the FM (9.63% d.b.) group. With the salted semi-drying method, fish muscle and fat can take up salt and lose water [33]. However, salinity is one of the risk factors for developing cardiovascular diseases, including hypertension [34].

Salt-treated fish have higher salinity due to the penetration of salt into fat, and salt penetration increases with increasing preservation periods [35]. In this study, similar results were observed; the salinity of the SSDM-CON group was 1.78%, significantly (*p* < 0.05) higher than that of the FM (0.84%) or SSDM-SAL (1.19%) groups. The dry base salt content results also indicated that the SSDM-CON group had 5.44% dry base salt, which was significantly higher than the FM (2.85% d.b.) and SSDM-SAL (3.7% d.b.) groups, while the water activity (a_w_) of the SSDM-SAL group (0.925) was significantly (*p* < 0.05) lower than that of the FM group (0.931) or the SSDM-CON (0.93) group due to the general reduction in a_w_ in all of the treated samples. These results indicate that proximate compositions, including the salinity and a_w_ of the SSDM-SAL group, could extend the shelf-life of fish without peroxidation or quality changes at high moisture levels, more than the SSDM-CON group. Moreover, glasswort (SAL) powder, as a natural ingredient, could be used as a salt replacement for improving shelf-life stability and producing healthier semi-dried mullets with lower sodium contents. Similar results were obtained by preparing healthier dry-cured ham using 20 g/Kg *Salicornia herbacea* L. product as a salt replacement and this also extended their shelf-life [15].

### 3.3. Chromaticity

**Figure 2:**
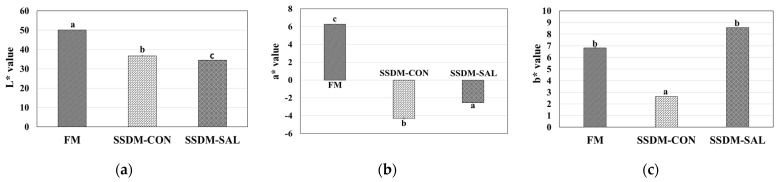
FM and SSDM groups color parameters of the L*, a*, and b* value changes are shown in (**a**–**c**), respectively. Comparisons were made between the fresh and experimental groups (*n* = 5 per group). The letters a, b and c in each bar graph represents significant difference at *p* < 0.05. L*, lightness; a*, redness; and b*, yellow.

Color is one of the important parameters affecting consumer acceptance [36]. Internal standards of color parameters L*, a*, and b* are used to measure food color [37]. The results of the color values are shown in Figure 2. The lightness (L*) of the FM sample was 50.1, which was significantly decreased to 36.7 and 34.5 in the SSDM-CON and SSDM-SAL groups, respectively, due to browning that occurred during the semi-dried salting process. The decoloration and browning of the salted semi-dried fish in the SSDM groups were due to enzymatic and non-enzymatic factors. The major cause of color changes is the formation of a brown fluorescent product that crosslinks with protein and lipid components which was explained in the Maillard reaction [38]. The a* value of the FM group was 6.28, indicating that a red color had appeared. However, the a* values in both SSDM groups (SSDM-CON and SSDM-SAL) were negative, indicating that the red color was reduced. The a* value of the SSDM-SAL group was −2.55, which was higher than that of the SSDM-CON group (a*: −4.3). The b* value of the FM group was 6.82. It was decreased to 2.64 in the SSDM-CON group. However, the b* value of the SSDM-SAL group was 8.56, which was higher than those of the FM and SSDM-CON groups. These color changes might have been due to changes of the fish muscles during the salted and semi-dried process. Lipid oxidation might also have played a role. In these processes, NaCl can promote lipid oxidation reactions between oxygen and free radicals in the presence of heat and/or light. Subsequently, the formation of metmyoglobin occurs in fish meat, promoting decoloration [39].

### 3.4. Changes in the Peroxide (PV), acid (AV), and Thiobarbituric Acid (TBARS) Values during Refrigerated Storage

**Figure 3:**
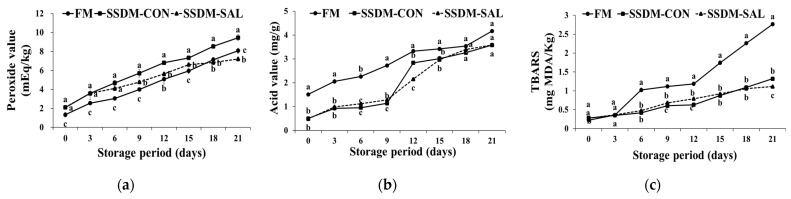
Changes in peroxide (PV) (**a**), acid (AV) (**b**), and TBARS value (**c**) in the FM and SSDM groups during refrigerated storage at 4 °C for 21 days. Values represent mean ± standard deviation (SD) (*n* = 5). Different superscript letters indicate significant differences (*p* < 0.05).

The PV, AV, and TBARS values indicated the inception of lipid oxidation during the ripening of the SSDMs. Changes in the PV, AV, and TBARS values of the FM and SSDM groups during 21 days storage in the refrigerator at 4 °C are shown in Figure 3. PV is an indicator of primary lipid oxidation and malodor formation [12]. The experimental results indicated that the initial PV of the FM and SSDM groups was low (1.33–2.1 mEq/kg), indicating the good quality of the product in terms of lipid oxidation. The PV values were significantly increased in all of the experimental groups during refrigerator storage for 21 days. A smaller increase in PV value was found for the FM group (from 1.33 to 8.08 mEq/kg); the SSDM-CON group showed a greater increase in PV value (from 2.1 to 9.45 mEq/kg); while the SSDM-SAL group’s PV value increased less (from 2.11 to 7.2 mEq/kg), than the FM and SSDM-CON groups during refrigerated storage, as shown in Figure 3a. The increased PV was caused by the oxidative degradation of the phospholipids, which were exposed to free radical toxicity, leading to cell damage [40]. However, when compared to the SSDM groups, the FM group demonstrated lower PVs over 15–18 days storage time, after which the PV levels significantly increased to more than the SSDM-SAL group over 21 days storage time. Moreover, the SSDM-CON group had higher PV levels than the SSDM-SAL group during the storage period, from 6 to 21 days, because the blood and other organic matter was spread over the sample surface, which might have activated peroxidase in the presence of salt and oxygen. However, the addition of SAL could suppress the PV and extend the shelf-life by inhibiting free radical toxicity when compared to the FM and SSDM-CON groups during the storage period. Kim et al. [41] reported that salt content has a high capability for promoting the generation of peroxides. However, lipid oxidation generally produces odors, including undesirable flavors, changing color, and so on.

In addition to the PV, the AV was measured to determine the degree of lipid hydrolysis in the FMs and SSDMs. Samples with increases in peroxide and acid values are liable to rapid oxidation during salting, drying, and storage under abused impelling conditions. The results in Figure 3b indicate that the FM AV values increased the most, from 1.51 mg/g to 4.17 mg/g. In the SSDM-CON group, it increased from 0.48 mg/g to 3.58 mg/g, and the SSDM-SAL group showed the lowest increase, from 0.5 mg/g to 3.58 mg/g during refrigerated storage. Falade and Oboh [42] suggested that the AVs gradually increased along with the storage period due to the deterioration, or rancidity, of the fish oil in the experimental groups. A better, higher-quality sample may feature lower acid values. Increased acidity due to the release of free fatty acids by lipid hydrolysis in the FM and SSDM samples may have led to the production of an off-odor, which can shorten the shelf-life of food. It was demonstrated that the formation of polyunsaturated fatty acids in muscle tissues is caused by oxidative deterioration. These results elucidate that the semi-dried mullets treated with glasswort (SAL) showed slightly decreased AVs although these were not significantly different to the FM and SSDM-CON groups. TBARS analysis is one of the most important methods for lipid oxidation analysis in fish species. TBARS is an indicator for identifying secondary oxidative aldehyde products via its reaction with malondialdehyde (MDA), and it can be used for determining the quality of refrigerated or chilled fish [43].

Fish products containing lipids and unsaturated fatty acids are decomposed via lipolytic and lipoxidative enzyme reactions in the presence of oxygen during the storage period. Peroxides are produced as primary products of fat oxidation and aldehydes, ketones, including short-chain fatty acids, are produced as secondary products of the fat oxidation. Thus, fish products might become rancid due to the oxidation of fats, which generates unwanted odor [44]. Oxidative rancidity is generally associated with MDA levels in food. Therefore, the oxidative rancidity was analyzed by measuring TBARS levels [45]. Taskaya and Yasar [46] suggested that TBA levels of less than 3 mg of MDA/kg of fish product are considered a very good condition. A TBA level from 3 mg up to 5 mg of MDA/kg is also considered a good condition; a range between 7 to 8 mg of MDA/kg of TBA is acceptable; and higher levels than 8 mg of MDA/kg indicate that the material is not good. By contrast, Wood and Enser [47] suggested that TBARS levels of less than 0.5 mg of MDA/kg of fish product are considered a good condition. A TBARS level of 1 mg of MDA/kg indicates a level of lipid oxidation that produces a rancid odor and taste that can be detected by consumers. TBARS levels higher than 2 mg of MDA/kg indicate that the material is likely to be detected as rancid by consumers, as well as producing other abnormal flavors [47].

However, in the current study, the results in Figure 3c indicate that the TBARS levels in the FM group reached up to 1 mg MDA/kg over a 6 day period. These levels reached up to 2.77 mg MDA/kg during 21 days refrigerated storage. This suggested that FMs produce rancidity and abnormal flavor when stored for more than a week, based on the study by Wood and Enser [47]. However, in the SSDM-CON and SSDM-SAL groups, values below 0.5 mg MDA/kg were observed during 7 days storage, and the value slightly increased, up to 1 mg MDA/kg, in 17–18 days. Subsequently, the SSDM-CON group’s MDA levels (1.4 mg MDA/kg) showed a greater increase than the SSDM-SAL group (1.11 mg MDA/kg). This indicates that, both the SSDM-CON and the SAL groups had lower TBARS levels up to 17–18 days; after that, and without SAL treatment, the rancidity in the SSDM-CON group drastically increased, according to the Wood and Enser categorization [47]. Cyprian et al. [48] also found a gradual increase in TBARS value with increasing storage periods in salted and dried capelin. The results indicate that processed foods with SAL (SSDM-SAL) treatment extended their shelf-life more than the SSDM-CON and FM groups during the storage period. The interaction between the variables of the mullet groups (FM, SSDM-CON and SSDM-SAL) and the storage time makes it possible to identify significant differences in PV, AV and TBARS levels during refrigerated storage. The two-way statistical ANOVA results strongly suggested that there was a statistically significant effect of the interaction between the mullet groups and storage time on PV (F = 7.394, *p* < 0.05), AV (F = 40.982, *p* < 0.05), and TBARS levels (F = 768.573, *p* < 0.05), which varied between the FM, SSDM-CON, and SSDM-SAL groups. These analyses results suggested that SAL treatment prevented PV, AV and TBARS levels and extended their shelf-life.

### 3.5. Fatty Acid Compositions

Fatty acids undergo important changes during semi-dried processing. These changes can significantly influence the nutritional properties of the final products. In this study, we observed the saturated fatty acids (SFAs), monounsaturated fatty acids (MUFAs), and polyunsaturated fatty acids (PUFAs). FMs and SSDMs have abundant fatty acids such as myristic acid (C14:0), palmitoleic acid (C16:1n-7), palmitic acid (C16:0), heptadecanoic acid (C17:0), stearic acid (C18:0), oleic acid (C18:1n-9), eicosapentaenoic acid (EPA, C20:5n-3), and docosahexaenoic acid (DHA, C22:6n-3) [49].

The results are shown in Table 2. These indicate that the FMs had the most abundant SFAs (approximately 50.8%), followed by PUFAs (32.98%), and MUFAs (16.21%). The salted semi-dried SSDM-CON group showed a higher percentage of MUFAs than the FM and SSDM-SAL groups. Palmitoleic acid (C16:1) is the major MUFA content in both the FM and SSDM groups. The PUFA contents were higher in the SSDM-SAL (41.39%) group than in FM (32.98%) and SSDM-CON (30.92%) groups. The SSDM-CON group had decreasing PUFA contents, specifically EPA and DHA levels, which might have been due to the structural and chemical changes that occurred during the fish drying process, resulting in the oxidation of lipids, thus decreasing the nutritional value of the fish [50]. With SAL treatment in the SSDM-SAL group, EPA and DHA based omega-3 fatty acids were increased. These EPA and DHA fatty acids can lower blood pressure and heart rate, thus improving blood vessel functions. Omega-3 fatty acids may also reduce cholesterol and triglyceride levels in the blood, as well as reduce hypertension, cardiovascular disease risk and they can also prevent human coronary artery disease [51].

The PUFA to SFA ratio can be used for analyzing the nutritional quality of fatty food materials. Health guidelines recommend that the PUFA to SFA ratio should be above 0.4 for a healthy diet [52]. This study shows that all of the treatments presented PUFA to SFA ratios of more than 0.4. However, in the SSDM-CON group, this ratio was 0.62, which was slightly lower than in the FM group (0.65), while the SSDM-SAL group showed a higher ratio (0.85) than both the FM and SSDM-CON groups. These results indicate that using SAL can enhance the nutritional value of processed mullets.

### 3.6. Amino Acid Compositions

Amino acid analysis is a key factor for measuring the nutritional value of processed food, which is also an important taste component of fish [53]. The total amount of amino acids in FMs was 19.31 g/100 g of muscle proteins, which was significantly higher than that of the SSDM-CON group (17.94 g/100 g) or the SSDM-SAL group (16.65 g/100 g), as shown in Table 3. Commonly, high amino acids levels are present in protein-rich foods. The results showed that the contents of aspartic acid, threonine, serine, glutamic acid, proline, glycine, alanine, cysteine, valine, methionine, isoleucine, leucine, tyrosine, phenylalanine, lysine and arginine were significantly lower in the SSDM groups than in the FM group. This might have been because amino acids are transferred from the tissues to the solution during the salting process, leading to a loss of nutrients [54]. In the case of histidine, its content was significantly more increased in the SSDM-SAL group than in the FM group. Similar results have been observed in Cecina slated and dried meat [55]. However, the contents of glutamic acid, a flavor-related ingredient, were approximately 16.02~16.32% in the FM and SSDM groups, indicating that all of the groups would have a similar meat flavor. The contents of essential amino acids ranged from 5.4% to 11.3%. They were lower in both the SSDM-CON and SSDM-SAL groups than in the FM group. These results indicate that SSDMs using SAL could enhance their refrigerated life with a smaller loss of constituent amino acids.

The total amount of free amino acids was 0.47 g/100 g in the fresh mullets. However, the liberation of peptides and free amino acids in the FM group was significantly lower than in the SSDM-CON group (0.56 g/100 g) or the SSDM-SAL group (0.55 g/100 g), as shown in Table 4. The SSDM-CON group showed the highest amount of free amino acids present in taurine, aspartic acid, threonine, serine, glutamic acid, glycine, alanine, valine, methionine, isoleucine, leucine, tyrosine, and lysine. This might have been due to protein degradation during the salted semi-dried treatment. Similar results have been reported during different salting and drying processes for yellow croaker (*Larimichthys polyactis*) [56]. By contrast, the arginine contents were drastically decreased in the SSDM-CON group compared with the FM and SSDM-SAL groups. Taurine-free amino acid accounted for approximately 37.36~44.03% of the total free amino acids in all the experimental groups, including the control. However, the liberation of peptides and free amino acids can occur in the proteolysis process during the ripening of salted fish [57]. These results indicated that the SSDM-SAL group liberated significantly fewer free amino acids than the FM group, although they were not significantly different numbers when compared with SSDM-CON group. 

### 3.7. SDS-PAGE

**Figure 4:**
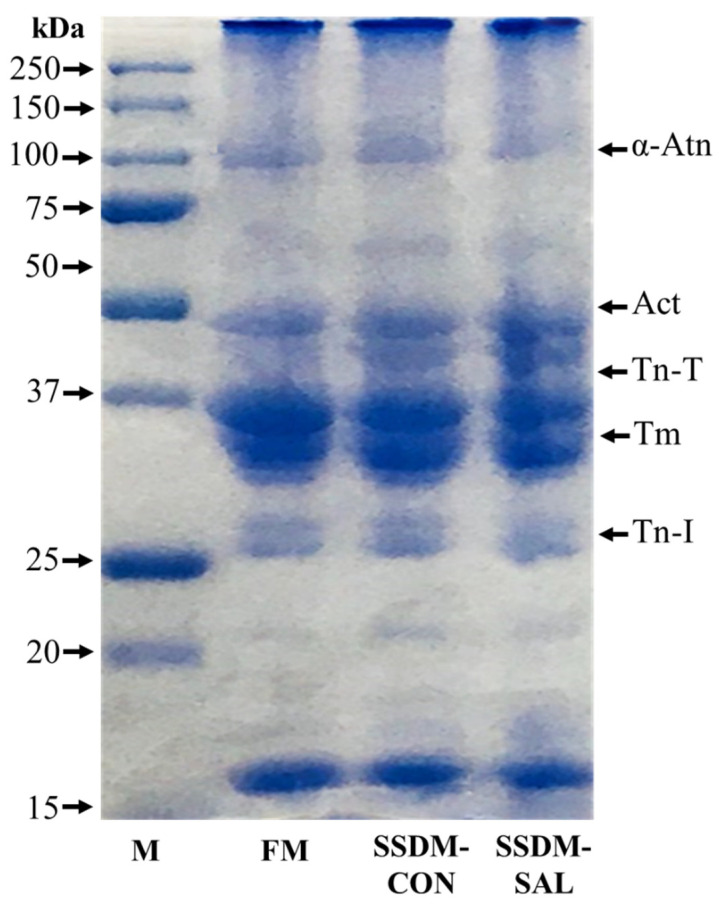
SDS-PAGE patterns of broad range protein marker (M), fresh mullet (FM), salted semi-dried mullet control (SSDM-CON) and salted semi-dried mullet with added SAL (SSDM-SAL) samples. The molecular weights of proteins are shown in kDa.

Protein levels of around 60–70% are present in the muscle fibers of fish meat [12]. The protein patterns in the FM and SSDM experiments were analyzed using SDS-PAGE electrophoresis. Major protein bands of α-actinin (α-Atn), actin (Act), troponin T type 3b protein fragment (Tn-T), tropomyosin (Tm), and troponin I protein (Tn-I) were observed in the FM and SSDM groups, as shown in Figure 4. These results indicate that there were no remarkable changes in the protein profiles between the FM, SSDM-CON, and SSDM-SAL groups. However, intensities of actin and Tn-T bands were higher in the SSDM-SAL group than in the SSDM-CON and FM groups. This might be because the proteins in the SSDM-SAL group were less influenced by denaturation during the salted and semi-dried process.

Previous studies have reported that the number of protein bands in Surimi-Crab meat samples is decreased during the salting and drying process. Moreover, the number of high-molecular-weight proteins is drastically decreased compared with that of medium-molecular-weight protein [58]. Such changes to proteins might be caused by salt because salt can increase protein solubility. Salted and dried fish can lose their flavor due to the loss of soluble and volatile flavor proteins [59]. However, the results of the current study revealed that the salted and semi-dried process with SAL treatment produced better results than the salting and drying method alone for preventing the loss of protein and for extending the shelf-life with better nutritional values.

### 3.8. Changes in Total Bacteria, Coliform, Escherichia coli, Vibrio parahaemolyticus and Staphylococcus aureus Levels during the Storage Period

The total number of bacteria can be used as an indication of quality and appropriation during fish storage; it can also be used to estimate the degree of bacterial damage [60]. Fish damage can occur due to physical and chemical changes. The reason for such damages or changes depends on microbial growth, which is influenced by factors such as fish contamination during salting and drying. In general, due to the nature of seafood, the water content is very high, which encourages rapid decay due to the growth of microorganisms.

**Figure 5:**
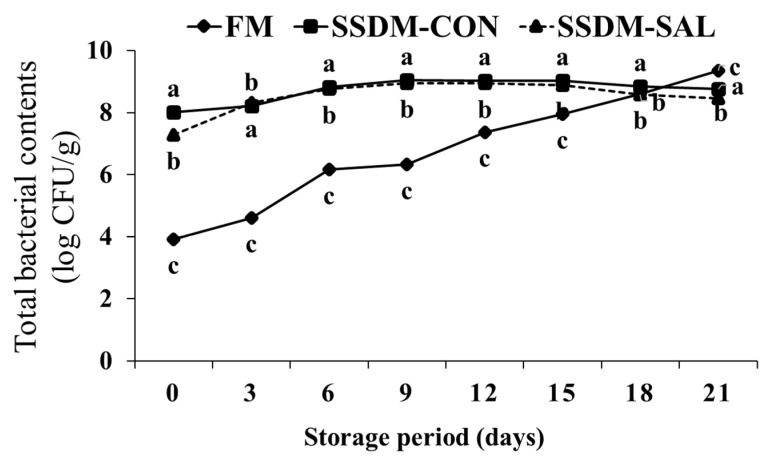
Changes in total microbial counts (log CFU/g) of salted semi-dried mullet during storage at 4 °C for 21 days. Values are presented as mean ± standard deviation (SD) (*n* = 5). The different superscript letters within each column indicate significant differences (*p* < 0.05).

As shown in Figure 5, during refrigerated storage (4 °C) for 21 days, the total microbial counts gradually increased from 3.92 to 9.34 log CFU/g in the FM group. The SSDM groups had higher initial microbial levels than the FM group. The total microbial counts increased from 8.01 to 8.76 log CFU/g in the SSDM-CON group and 7.28 to 8.46 log CFU/g in the SSDM-SAL group. The decrease in the total bacterial counts was expected due to the semi-dried salting process in the SSDM groups. The total bacterial contents were significantly increased in both the FM and the SSDM groups. This might be because bacteria adapted to the fish skin environment, the major source of the development of microbial organisms during storage [35]. Other studies have suggested that processed fish products might be contaminated by microorganisms during fish processing and storage [61]. However, during storage, the total bacterial count rapidly increased in the FM group; moreover, the count was significantly higher after 18 days storage time when compared to the SSDM-CON and SSDM-SAL groups. In addition, in the SSDM-CON group, the microbial count was significantly higher than in the SSDM-SAL group. This indicates that the SAL treatment improved microbial safety during the refrigerated storage time. This demonstrates that bioactive compounds were present in the SAL, which showed antioxidant and antimicrobial effects. The two-way statistical ANOVA results strongly suggested that there was a statistically significant effect of the interaction between the mullet groups and the storage time on the total bacterial content (F = 937.149, *p* < 0.05), which varied between the FM, SSDM-CON and SSDM-SAL groups during storage. These results suggested that the SAL treatment prevented bacterial growth in the SSDM-SAL group compared with the FM and SSDM-CON groups.

Coliform and *E-coli* measurement is a good indicator of the hygienic condition of a product during fish processing. In general, food is susceptible to microorganisms; thus, care must be taken in the distribution, processing, and storage (including storage areas) stages to prevent microbial contamination [61]. As shown in Table 5, the coliform level was 4.23 log CFU/g in the FM group. It was higher in the SSDM-CON group (4.72 log CFU/g). SAL treatment significantly decreased the coliform levels to 4.45 log CFU/g in the SSDM-SAL group. Similar results have been reported for raw fish material (2 ± 0 log CFU/g), reaching 5 ± 0 log CFU/g on the 15th day in the salt-treated group [35]. However, *E-coli* bacteria were not detected in the FM or SSDM groups.

*Vibrio* spp. is a pathogenic microorganism that causes major food poisoning, which can infect both fish and shellfish. It is necessary to analyze *Vibrio* spp. before the consumption of food for safety reasons [62]. The International Association of Microbiology Society has suggested that fresh and frozen fish should not contain *Vibrio* spp. In the present study, enteritis vibrio was not detected in any of the samples, as shown in Table 5, indicating that the FM and SSDM samples were of good quality.

*Staphylococcus aureus*, a well-known foodborne pathogen, can be found even under adverse environmental conditions. Foods that are rich in protein and carbohydrates are highly likely to be contaminated by *S. aureus*, which causes food poisoning [63]. The results of the present study showed that none of the experimental groups (FM and SSDM) had *Staphylococcus aureus*, as shown in Table 5. This indicates that the semi-dried salted method using SAL treatment for preservation can prevent microbial contamination and extend their shelf-life.

## 4. Conclusions

This study investigated the effects of processing methods on the quality and nutritional characteristics of salted semi-dried mullets treated with SAL after long-term refrigerated storage. The antioxidant activity of *S. herbacea* L. treatment strongly inhibited DPPH and ABTS radicals at 2 mg/mL concentrations, which reduced fatty acid oxidation and rancidity by suppressing the PV, AV and TBARS levels as well as by protecting against microorganisms during refrigerated storage. It was found that SAL treatment significantly inhibited the PV and TBARS levels after 21 days storage time, when compared with the FM and SSDM-CON groups. In addition, SAL treatment maintained the nutritional values of the mullets by decreasing the denaturation of crude proteins and fats, including essential PUFAs and omega-3 fatty acids, during refrigerated storage. This indicates that, when compared with the fresh and salted control groups, SAL treatment prevented lipid peroxidation and rancidity to extend the mullets’ shelf-life. However, the total microbial count significantly increased during storage time in the FM group; these levels were higher than in the SSDM groups during 21 days refrigerated storage. Additionally, SAL treatment inhibited the microorganism count after 21 days when compared with the FM and SSDM-CON groups. This may have been because the fresh mullets were spoiled by microbial effects over a short time period. However, treatment with 0.2% of *S. herbacea* L. extended their shelf-life by preventing microorganism growth in the SSDM-SAL group compared with the FM and SSDM-CON groups. Moreover, increased concentrations of SAL treatment may provide a salt replacement according to previous studies, irrespective of meat color. This is an important limitation of this study; using a lower amount (0.2%) of SAL treatment may improve the antioxidant properties as well as the meat color, which is important for consumer acceptance.

## Figures and Tables

**Table 1:** Proximate composition, salinity, and water activity of the FM and SSDM groups.

Groups	Proximate Composition					
	Moisture (%)	Ash (% fw)	Crude Fat (% fw)	Crude Protein (% fw)	Salinity (%)	Water Activity
FM	70.61 ± 0.36 ^a^	1.30 ± 0.09 ^b^	2.83 ± 0.03 ^c^	22.95 ± 0.09 ^c^	0.84 ± 0.03 ^c^	0.93 ± 0.01 ^a^
SSMD-CON	67.31 ± 0.36 ^c^	2.28 ± 0.09 ^a^	3.24 ± 0.02 ^b^	24.59 ± 0.27 ^b^	1.78 ± 0.03 ^a^	0.93 ± 0.01 ^a^
SSDM-SAL	68.03 ± 0.18 ^b^	2.32 ± 0.09 ^a^	3.18 ± 0.04 ^a^	25.68 ± 0.09 ^a^	1.19 ± 0.02 ^b^	0.92 ± 0.01 ^b^

Values represent mean ± standard error (SE) (*n* = 5). Different superscript letters within each column represent significant differences (*p* < 0.05). Fresh mullet (FM); salted semi-dried mullet control (SSDM-CON); salted semi-dried mullet with added SAL (SSDM-SAL).

**Table 2:** Fatty acid compositions (g/100 g total fatty acids) of FM and SSDM groups.

Fatty Acids	Experimental Groups		
	FM	SSDM-CON	SSDM-SAL
Butyric acid (C4:0)	-	-	-
Caproic acid (C6:0)	-	-	-
Caprylic acid (C8:0)	-	-	-
Capric acid (C10:0)	-	-	-
UNDecanoic acid (C11:0)	-	-	-
Luric acid (C12:0)	-	-	-
Tridecanoic acid (C13:0)	-	-	-
Myristic acid (C14:0)	3.41 ± 0.03 ^a^	3.17 ± 0.01 ^b^	2.10 ± 0.01 ^c^
Pentadecanoic acid (C15:0)	1.26 ± 0.02 ^a^	1.19 ± 0.10 ^a^	0.60 ± 0.03 ^b^
Palmitic acid (C16:0)	16.22 ± 0.85 ^a^	14.99 ± 1.36 ^a^	12.56 ± 1.23 ^b^
Heptadecanoic acid (C17:0)	23.41 ± 1.94 ^a^	22.45 ± 1.47 ^a^	25.25 ± 2.47 ^a^
Stearic acid (C18:0)	5.69 ± 0.06 ^c^	6.93 ± 0.15 ^a^	6.43 ± 0.02 ^b^
Arachidic acid (C20:0)	-	-	-
Heneicosanoic acid (C21:0)	-	-	-
Behenic acid (C22:0)	-	-	-
Tricosanoic acid (C23:0)	0.81 ± 0.01 ^c^	0.99 ± 0.01 ^b^	1.41 ± 0.01 ^a^
Lignoceric acid (C24:0)	-	-	-
Total saturated fatty acid (SFAs)	50.80 ± 2.91 ^a^	49.72 ± 3.10 ^a^	48.35 ± 3.77 ^a^
Myristoleic acid (C14:1)	-	-	-
cis-10-Pentadecenoic acid (C15:1)	-	-	-
Palmitoleic acid (C16:1)	10.39 ± 0.74 ^b^	12.66 ± 1.32 ^a^	6.65 ± 0.61 ^c^
cis-10-Heptadecenoic acid (C17:1)	-	-	-
Elaidic acid (C18:1n9t)	-	-	-
Oleic acid (C18:1n9c)	5.82 ± 0.21 ^b^	6.70 ± 0.25 ^a^	3.61 ± 0.25 ^c^
cis-11-Eicosenoic acid (C20:1)	-	-	-
Erucic acid (C22:1n9)	-	-	-
Nervonic acid (C24:1)	-	-	-
Total monounsaturated fatty acid (MUFAs)	16.21 ± 0.84 ^b^	19.36 ± 1.33 ^a^	10.26 ± 0.54 ^c^
Linolelaidic acid (C18:2n6t)	-	-	-
Linoleic acid (C18:2n6c)	-	-	-
cis-11,14-Eicosadienoic acid (C20:2)	-	-	-
cis-13,16-Docosadienoic acid (C22:2)	-	-	-
ɣ-Linolenic acid (C18:3n6)	-	-	-
Linolenic acid (C18:3n3)	-	-	-
cis-8, 11, 14-Eicosatrienoic acid (C20:3n6)	-	-	-
cis-11,14,17-Eicosatienoic acid (C20:3n3)	-	-	-
Arachidonic acid (C20:4n6)	-	-	-
cis-5,8,11,14,17-Eicosapentaenoic acid (C20:5n3)	28.29 ± 1.61 ^b^	26.62 ± 1.75 ^b^	36.00 ± 2.11 ^a^
cis-4,7,10,13,16,19-Docosahexaenoic acid (C22:6n3)	4.70 ± 0.09 ^b^	4.30 ± 0.01 ^c^	5.39 ± 0.11 ^a^
Total polyunsaturated fatty acid (PUFAs)	32.98 ± 1.92 ^b^	30.92 ± 2.18 ^b^	41.39 ± 1.11 ^a^
PUFA to SFA ratio	0.65	0.62	0.86

Note: ‘-’: represents that the corresponding fatty acid is not detected. Values are presented as mean ± standard deviation (SD) (*n* = 5). The different superscript letters within each row indicate significant differences (*p* < 0.05). Fresh mullet (FM); salted semi-dried mullet control (SSDM-CON); salted semi-dried mullet with added SAL (SSDM-SAL).

**Table 3:** Constituent amino acids (g/100 g) of FM and SSDM groups.

Amino Acids	Experimental Groups		
	FM	SSDM-CON	SSDM-SAL
Aspartic acid	1.95 ± 0.02 ^a^	1.82 ± 0.02 ^b^	1.70 ± 0.02 ^c^
Threonine	0.94 ± 0.04 ^a^	0.89 ± 0.04 ^ab^	0.82 ± 0.02 ^b^
Serine	0.86 ± 0.02 ^a^	0.81 ± 0.02 ^b^	0.74 ± 0.02 ^c^
Glutamic acid	3.15 ± 0.03 ^a^	2.87 ± 0.04 ^b^	2.67 ± 0.04 ^c^
Proline	0.81 ± 0.01 ^a^	0.72 ± 0.02 ^b^	0.67 ± 0.02 ^c^
Glycine	1.09 ± 0.02 ^a^	0.91 ± 0.02 ^b^	0.84 ± 0.03 ^c^
Alanine	1.25 ± 0.02 ^a^	1.18 ± 0.05 ^b^	1.07 ± 0.03 ^c^
Cystine	0.08 ± 0.01 ^a^	0.05 ± 0.00 ^b^	0.05 ± 0.03 ^b^
Valine	0.99 ± 0.02 ^a^	0.92 ± 0.02 ^b^	0.87 ± 0.02 ^c^
Methionine	0.66 ± 0.02 ^a^	0.64 ± 0.02 ^a^	0.54 ± 0.02 ^b^
Isoleucine	0.90 ± 0.03 ^a^	0.86 ± 0.02 ^a^	0.79 ± 0.01 ^b^
Leucine	1.66 ± 0.02 ^a^	1.59 ± 0.04 ^b^	1.45 ± 0.02 ^c^
Tyrosine	0.74 ± 0.03 ^a^	0.73 ± 0.02 ^a^	0.65 ± 0.02 ^b^
Phenylalanine	0.83 ± 0.02 ^a^	0.82 ± 0.03 ^a^	0.75 ± 0.01 ^b^
Histidine	0.52 ± 0.02 ^a^	0.52 ± 0.02 ^a^	0.55 ± 0.02 ^b^
Lysine	1.76 ± 0.01 ^a^	1.57 ± 0.04 ^b^	1.54 ± 0.04 ^b^
Arginine	1.12 ± 0.02 ^a^	1.04 ± 0.03 ^b^	0.95 ± 0.04 ^c^
Total	19.31 ± 0.35 ^a^	17,94 ± 0.47 ^b^	16.65 ± 0.39 ^c^

Values are presented mean ± standard deviation (SD) (*n* = 5). The different superscript letters within each row indicate significant differences (*p* < 0.05). Fresh mullet (FM); salted semi-dried mullet control (SSDM-CON); salted semi-dried mullet with added SAL (SSDM-SAL).

**Table 4:** Free amino acids (g/100 g) of FM and SSDM groups.

Free Amino Acids	Experimental Groups		
	FM	SSDM-CON	SSDM-SAL
Phosphoserine	-	-	-
Taurine	0.20 ± 0.01 ^a^	0.21 ± 0.00 ^a^	0.20 ± 0.00 ^a^
Phosphoethanolamine	-	-	-
Urea	-	-	-
Aspartic acid	0.01 ± 0.00 ^b^	0.01 ± 0.00 ^a^	0.01 ± 0.00 ^c^
Hydroxyproline	-	-	-
Threonine	0.02 ± 0.00 ^ab^	0.02 ± 0.00 ^b^	0.02 ± 0.00 ^c^
serine	0.02 ± 0.00 ^b^	0.03 ± 0.00 ^a^	0.01 ± 0.00 ^c^
Asparagine	-	-	-
Glutamic acid	0.01 ± 0.00 ^b^	0.03 ± 0.00 ^a^	0.01 ± 0.00 ^b^
Sarcocine	-	-	-
α-aminoadipic acid	-	-	-
Proline	-	-	-
Glycine	0.05 ± 0.00 ^c^	0.07 ± 0.00 ^a^	0.07 ± 0.00 ^b^
Alanine	0.07 ± 0.00 ^b^	0.07 ± 0.00 ^a^	0.04 ± 0.0 ^c^
Citrulline	-	-	-
α-aminobutyric acid	-	-	-
Valine	0.01 ± 0.00 ^b^	0.01 ± 0.00 ^a^	0.01 ± 0.00 ^c^
Cystine	-	-	-
Methionine	0.01 ± 0.00 ^a^	0.01 ± 0.00 ^a^	0.005 ± 0.00 ^b^
Isoleucine	0.01 ± 0.00 ^b^	0.01 ± 0.00 ^a^	0.005 ± 0.00 ^c^
Leucine	0.01 ± 0.00 ^b^	0.03 ± 0.00 ^a^	0.01 ± 0.00 ^c^
Tyrosine	0.01 ± 0.00 ^c^	0.01 ± 0.00 ^a^	0.01 ± 0.00 ^b^
phenylalanine	-	-	-
β-alanine	-	-	0.02 ± 0.00 ^NS^
β-aminoisobutyric acid	-	-	-
γ-amino-n-butyric acid	-	-	-
Histidine	-	-	0.09 ± 0.00 ^NS^
1-methylhistidine	-	-	-
3-methylhistidine	-	-	-
Carnosine	-	-	-
Tryptopan	-	-	-
Hydroxylysine	-	-	-
Ornitine	-	-	0.01 ± 0.00 ^NS^
Lysine	0.03 ± 0.00 ^b^	0.04 ± 0.00 ^a^	0.02 ± 0.00 ^b^
Arginine	0.01 ± 0.00 ^a^	0.01 ± 0.00 ^c^	0.01 ± 0.00 ^b^
Total	0.47 ± 0.02 ^b^	0.56 ± 0.02 ^a^	0.55 ± 0.02 ^a^

Note: ‘-’, corresponding amino acid was not detected. Values are presented as mean ± standard deviation (SD) (*n* = 5). The different superscript letters within each row indicate significant differences (*p* < 0.05), superscripted NS = not significant. Fresh mullet (FM); salted semi-dried mullet control (SSDM-CON); salted semi-dried mullet with added SAL (SSDM-SAL).

**Table 5:** Total coliforms, *E. coli*, *Vibrio* and *Staphylococcus* contents (log CFU/g) in FM and SSDM groups.

Groups	Coliforms	*E. coli*	*Vibrio parahaemolyticus*	*Staphylococcus aureus*
FM	4.23 ± 0.02 ^c^	ND	ND	ND
SSDM-CON	4.72 ± 0.01 ^a^	ND	ND	ND
SSDM-SAL	4.45 ± 0.02 ^b^	ND	ND	ND

Values are presented as mean ± standard deviation (SD) (*n* = 5). The different superscript letters within each column indicate significant differences (*p* < 0.05). ND, not detectable (level less than 1 log cfu/g). Fresh mullet (FM); salted semi-dried mullet control (SSDM-CON); salted semi-dried mullet with added SAL (SSDM-SAL).

## Data Availability

Not applicable.
